# The Impact of Inorganic Nitrate on Endothelial Function: A Systematic Review of Randomized Controlled Trials and Meta-analysis

**DOI:** 10.1093/nutrit/nuaf132

**Published:** 2025-07-18

**Authors:** Begum Celik, Esther Muriuki, Gunter G C Kuhnle, Jeremy P E Spencer, Charlotte E Mills

**Affiliations:** Hugh Sinclair Unit of Human Nutrition, School of Chemistry, Food and Nutritional Sciences, University of Reading, Reading RG6 6AP, United Kingdom; Section of Nutrition, Faculty of Medicine, Imperial College London, London W12 ONN, United Kingdom; Hugh Sinclair Unit of Human Nutrition, School of Chemistry, Food and Nutritional Sciences, University of Reading, Reading RG6 6AP, United Kingdom; Hugh Sinclair Unit of Human Nutrition, School of Chemistry, Food and Nutritional Sciences, University of Reading, Reading RG6 6AP, United Kingdom; Hugh Sinclair Unit of Human Nutrition, School of Chemistry, Food and Nutritional Sciences, University of Reading, Reading RG6 6AP, United Kingdom; Hugh Sinclair Unit of Human Nutrition, School of Chemistry, Food and Nutritional Sciences, University of Reading, Reading RG6 6AP, United Kingdom

**Keywords:** inorganic nitrate, beetroot, endothelial function, flow-mediated dilation, FMD, cardiovascular disease

## Abstract

**Context:**

Inorganic nitrate is an exogenous source of nitric oxide, an established regulator of vascular homeostasis via the nitrate-nitrite-nitric oxide pathway. Here, we evaluate the impact of inorganic nitrate on endothelial function, a risk factor for cardiovascular disease.

**Objective:**

A systematic review of the existing literature and meta-analysis was performed. Trials testing inorganic nitrate compared with a control were selected and the change in forearm endothelial function (as assessed by flow-mediated dilatation [FMD]) were included.

**Data Sources:**

The following databases were searched: Medline, Web of Science, and Scopus.

**Data Extraction and Analysis:**

Standardized mean differences in %FMD were pooled using a random-effects model and 13 studies were included in the analysis. Quality assessment was performed using the Cochrane risk-of-bias score.

**Results:**

Inorganic nitrate was associated with improved Δ%FMD compared with the control; the standardized mean difference was 1.48% (95% CI: 0.70%–2.27%; P < 0.01); high heterogeneity (I^2^ = 98.2%) was observed. The significant effect observed remained irrespective of dose (±600 mg nitrate), duration (acute or chronic consumption), health status (± health conditions), and type of nitrate (dietary or nondietary). Notably, acute studies had a greater FMD response than chronic studies (1.93% [95% CI: 0.71%–3.15%] vs 0.90% [95% CI: 0.48%–1.31%]). More than half of the studies reviewed showed low risk of bias; the rest of the studies were classified as “some concern” due to lack of information about randomization process and lack of prespecified analysis plans.

**Conclusion:**

Our results show that, from a modest number of included trials, inorganic nitrate consumption improves FMD response by more than 1%, a clinically relevant magnitude for the prevention of cardiovascular disease.

**Systematic Review Registration:**

PROSPERO registration no. CRD42020191832.

## INTRODUCTION

Cardiovascular disease (CVD) remains one of the major causes of global mortality and its burden continues to increase globally.^1^ Modifiable behaviors, such as diet, could play a key role in cost-effective public health strategies to reduce the risk of CVD. It is widely accepted that dietary approaches, such as the Dietary Approaches to Stop Hypertension (DASH) or Mediterranean diet, reduce CVD risk,^2^ but understanding more about the actions of individual components of these diets is imperative to make more-specific recommendations. Inorganic nitrate, which is high in green vegetables and beetroot, is likely to contribute to some of the beneficial effects observed with these dietary patterns.

The consumption of high-nitrate foods provides an exogenous source of nitric oxide (NO) via the nitrite-nitrate-nitric oxide (NO) pathway, whereby nitrate enters the entero-salivary circulation and is reduced to nitrite by commensal bacteria in the mouth.^3^^,^^4^ This nitrite enters the circulation where it is reduced further to NO.^5^^,^^6^ It is well established that NO is a vasodilator and there is a growing body of evidence that shows that consumption of inorganic nitrate reduces blood pressure (BP) by approximately 4 mm Hg,^7^ which is akin to many antihypertensive medications,^8^ demonstrating true potential for reducing CVD risk. The impact on other CVD risk factors is less established, although there is some evidence for a reduction in platelet aggregation^9^^,^^10^ and reduction in arterial stiffness^11^^,^^12^ and improvement in vascular structure and function.^13^ Although not regularly used in clinical practice, endothelial function, typically performed by flow-mediated dilatation (FMD), is an important predictor of CVD risk and prognosis.^14^

Herein, we present a systematic review and meta-analysis of randomized controlled trials (RCTs) investigating the efficacy of inorganic nitrate on endothelial function measured by FMD in adults. An investigative subanalysis is presented assessing the impact of duration of intervention, dose of nitrate, health status of participants, and the source of nitrate.

## METHODS

This systematic review and meta-analysis was conducted according to the PRISMA (Preferred Reporting Items for Systematic Reviews and Meta-Analyses) guidelines.^15^ The protocol is published in the International Prospective Register of Systematic reviews (PROSPERO ID: CRD42020191832). The research question, structured according to the PICOS (Participants, Intervention, Comparison, Outcome, and Study design) framework, is outlined in Table 1.

**Table 1.** PICOS Criteria for Inclusion and Exclusion of Studies

Parameter	Inclusion criteria	Exclusion criteria
Population	Adult males and females (>18 y) with or without health conditions	Population aged <18 y
Intervention	Oral administration of inorganic nitrate (dietary or supplement forms)	Research studies that investigated the action of drug-related products
Comparator	Nitrate-free control	—
Outcomes	Assessment of endothelial function by flow-mediated dilatation (FMD) on the brachial artery	—
Study design	Randomized controlled trials	Cohort and observational studies

### Eligibility Criteria

The publications considered for inclusion in this review were RCTs in adult (>18 years) male or female human participants. Trials in participants with and without health conditions were included. Only trials that tested the impact of inorganic nitrate in either dietary (eg, beetroot, spinach, or lettuce) or nondietary (eg, sodium nitrate or potassium nitrate) forms versus a control were included. Trials were only included if an outcome was endothelial function and change in percentage flow-mediated dilatation (%FMD) was presented (before vs after intervention) or could be calculated. Only trials that measured FMD at the brachial artery were included. Studies that co-administered nitrate with another intervention (eg, exercise, another dietary source, or drugs) were excluded. Trials that were not randomized or had no control were excluded; similarly, observational (eg, cross-sectional and cohort trials) were not included in this review. Trials were excluded where data were missing and could not be obtained from the authors. Subanalyses were classified in terms of the following: health status (healthy, with health conditions [eg, hypertension, diabetes, overweight/obese]), study duration (acute, defined as postprandial, single dose and/or <24 h in duration, or chronic), intervention type (nondietary or dietary sources of nitrate), inorganic nitrate dosage (low, ≥600 mg; high, <600 mg), and participants’ age (<50 years and ≥50 years). Where necessary, participants of the studies were classified by considering the following characteristics: body mass index (BMI; <30, ≥30 kg/m^2^) systolic BP (<140, ≥140 mmHg), diastolic BP (<90, ≥90 mmHg), and age (≥50, <50 years). The age cutoff of 50 years was chosen because it generally aligns with the onset of age-related cardiovascular risk factors. After the age of 50, the prevalence of CVD and endothelial dysfunction increases, likely due to changes in vascular physiology, hormonal shifts (especially in postmenopausal women), and accumulated lifestyle factors. The nitrate amount of 600 mg was chosen as it represents twice the effective dose commonly recognized in the literature. This level was selected to ensure a pronounced physiological response, while also highlighting the challenges of achieving such an intake through a typical diet. For instance, reaching this amount would require consuming approximately 5 portions of beetroot, which may not be practical for most individuals.

### Information Sources and Search Strategy

Three databases—Medline, Web of Science, and Scopus—were used to systemically identify relevant publications. Studies published before February 2023 were included and the review was restricted to studies published in English. Where data were missing, authors were contacted. Reference lists of the relevant articles were searched to check any potential additional studies that were not be found by the search strategy. Auto filters were used in terms of text availability (full text) article type (RCTs), and language (English).

Predefined terms were used for the search, using MeSH (Medical Subject Heading) terms where appropriate. Search terms included inorganic, nitrate, beetroot, endothelial, FMD, nitric oxide, NO, and vascular and search algorithms were created with specific building blocks (Boolean terms and truncation details of the search algorithm are presented in Table S1).

### Study Selection

Articles were assessed for eligibility by 2 authors independently (B.C. and E.M.). In the first phase, titles and abstracts were screened and those articles that potentially met the inclusion criteria were moved to phase 2, where full texts were reviewed. In the case of disagreement between reviewers in phase 1, the articles in question were moved to phase 2 by default. Where disagreement was at full-text review (phase 2), a third reviewer (C.E.M.) reviewed the articles and a decision was reached by consensus.

### Data Extraction

For each eligible trial, the following study characteristic details were extracted into a table: publication year, name of the first author, information of participants (sex, age, weight, BMI, health condition), design of the study (eg, parallel or crossover details of washout periods), intervention dose, duration, type of intervention, and control/placebo. In acute trials where FMD was measured multiple times, baseline and the time point falling between 2–3 hours after consumption of the intervention were used to coincide with the estimated peak nitrate absorption time, occurring between 2 and 3 hours, as peak time demonstrated in previous studies.^12^^,^^16^^,^^17^ For chronic trials, measurements at the end of the intervention period were taken. In the case of missing data, authors were contacted; where no data could be obtained, publications were excluded. For studies that involved multiple intervention arms, data were extracted from each relevant arm and compared with the control.

### Study Risk-of-Bias Assessment

Two independent reviewers (B.C. and E.M.) assessed the publications in terms of the risk of bias and the quality by using the Cochrane Risk of Bias Assessment version 2 (RoB 2).^18^ This tool considers bias from (1) randomization process, (2) deviations from the intended intervention, (3) missing data, (4) measurement of the outcome, and (5) selection of reported results. Studies were judged on each domain as low risk, some concerns, and high risk; and these results were used to calculate overall risk of bias. In the case that all domains were judged as low risk, the overall risk of bias was classified as such. If at least 1 domain was judged to have some concerns, but no domain had a high risk of bias, then the research was categorized as “some concerns.” If at least 1 domain was judged as high risk, then the research was categorized as such. Where there was disagreement between the assessors, a third assessor was involved (C.E.M.), and the decision for categorization was made by consensus. For the assessment of the studies, the full articles were used as a main source of information; in some incidences, the clinical registration information of the studies was checked.

### Statistical Analysis

R statistical software (R. 4.3.2; R Foundation for Statistical Computing, Vienna, Austria^19^) was used to perform the meta-analysis. First, for both intervention and control groups, differences in mean %FMD values were calculated by taking differences of the values between before and after nitrate administration. Second, using random-effects models, pooled summary estimates and differences in means (standardized mean difference [SMD]) were calculated between intervention and control groups. An estimate of heterogeneity in observed effects was reported as *I^2^*. This ranged between 0% and 100% and was classified as low (0%–25%), medium (25%–75%), and high (75%–100%) variance in effects.^18^

To evaluate publication bias and selective reporting bias, a funnel plot and Egger’s regression were performed. To assess the impact of excluding trials that might cause a high risk of bias, sensitivity analysis was performed. We performed 4 post hoc sensitivity analyses removing trials with (1) high nitrate dosage (doses >1000 mg), (2) food-based control (apart from nitrate-free beetroot juice), (3) large variance in Δ%FMD (defined as 95% CI of >1.5% FMD), and (4) having high Δ%FMD responses (>2 SDs from the mean).

## RESULTS

### Study Selection and Characteristics

**Figure 1.**
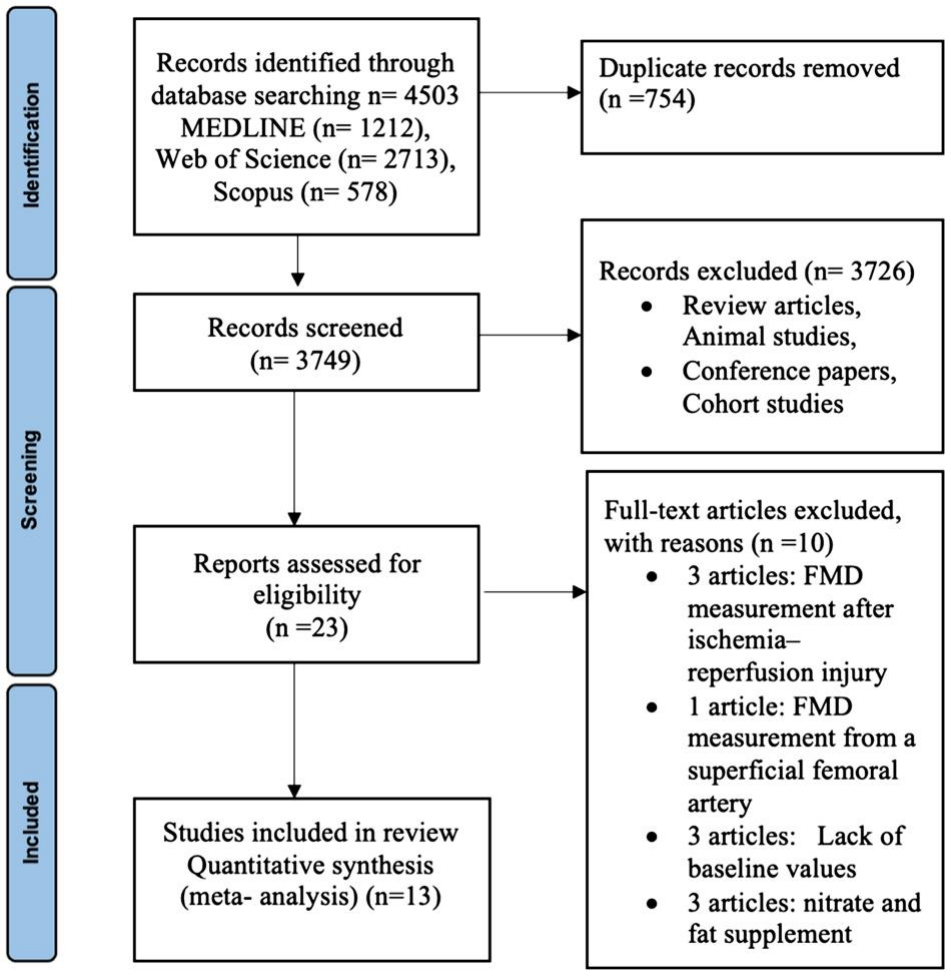
Flow Chart of the Literature Search. Abbreviation: FMD, flow-mediated dilatation

In total, 4503 studies were identified after the electronic search of the 3 databases, *n* = 754 duplicates were removed. Abstracts and titles of the remaining 3749 articles were screened. The remaining 23 publications were included for full-text review, and a further 10 were excluded. Thirteen studies with multiple eligible arms were included in the review and meta-analysis (Figure 1).

All included studies used a randomized, placebo-controlled design. Studies were completed between 2012 and 2022. The pooled sample size from the included studies was *n* = 380; the sample size for each trial ranged between *n* = 10 and *n* = 65.

The intervention duration of the acute studies varied from 1.5 to 4 hours, while for the duration of the chronic studies, this varied from 8 days to 6 weeks. Five studies used a parallel study design,^11^^,^^12^^,^^20–22^ whereas the remaining 8 studies used a crossover study design.^23–30^ Summaries of the included trials are presented in Tables 2 and 3.

**Table 2.** Characteristics of the Included Acute Studies

Study (year)	No. of participants	Population characteristics^a^	Study design	Study duration (washout^b^)	Active intervention	Nitrate dose in active arm	Control	Impact on %FMD
Heiss et al (2012)^23^	10	Healthy, 50% M, 26 ± 1.0 y	Crossover, RCT, double-blind	1.5 h	NaNO_3_	1058 mg	Water	↑
Kapil et al (2018) (1)^24^	13	Healthy, 100% F, 28 ± 1.7 y	Crossover, RCT, double-blind	3 h (7–28 d)	KNO_3_	809 mg (8 mmol)	KCl	↔
Kapil et al (2018) (2) ^24^	13	Healthy, 100% M, 28 ± 1.6 y	Crossover, RCT, double-blind	3 h (7–28 d)	KNO_3_	809 mg (8 mmol)	KCl	↔
Rodriguez-Mateos et al (2015) (1)^29^	10	Healthy, 100% M, 25 ± 1.6 y	Crossover, RCT, double-blind	2 h (7 d)	NaNO_3_	225 mg (3 mg/kg)^c^	Water	↑
Rodriguez-Mateos et al (2015) (2)^29^	10	Healthy, 100% M, 25 ± 1.0 y	Crossover, RCT, double-blind	2 h (7 d)	NaNO_3_	637 mg (8.5 mg/kg bw)^c^	Water	↑
Velmurugan et al (2016) (1)^11^	34/33^d^	Hypercholesterolemic (mean, 6.7 mmol/L), 54%/57% M, 53 ± 1.8/53.3 ± 2.1 y^d^	Parallel, RCT, double-blind	3 h	BRJ	375 mg (250 mL)	NDBJ	↑
Volino-Souza et al (2018)^28^	12	Pregnant, 100% F, 27 ± 2.3 y	Crossover, RCT, double-blind	2.5 h (7 d)	BRJ	555 mg (140 mL)	NDBJ	↑
Nogueira Soares et al (2020) (1)^30^	18	Healthy, 100% M, 27 ± 1.9 y	Crossover, RCT, double-blind	2 h (7 d)	BRJ	506 mg (140 mL)	NDBJ	↑
Nogueira Soares et al (2020) (2)^30^	13	HIV-positive, 85% M, 36 ± 2.8 y	Crossover, RCT, double-blind	2 h (7 d)	BRJ	506 mg (140 mL)	NDBJ	↑
Smeets et al (2022)^26^	18	Abdominally obese (BMI, 33.5 ± 1.2 kg/m^2^; waist circumference, 117 ± 2.4 cm), 100% M, 50 years^e^	Crossover, RCT, double-blind	4 h	KNO_3_	625 mg	KCl	↔

↔ indicates no significant difference; ↑ indicates significant increase.

a Data are presented as mean ± SEM.

b Where available.

c Presented as mean intake.

d Data are presented for active/control arms.

e Data for SEM not available.

Abbreviations: BRJ, beetroot juice; F, female(s); FMD, flow-mediated dilation; KCl, potassium chloride; KNO_3_, potassium nitrate; M, male(s); NaNO_3_, sodium nitrate; NDBJ, nitrate-depleted beetroot juice; RCT, randomized controlled trial.

**Table 3.** Characteristics of the Included Chronic Studies

Study (year)	No. of participants	Population characteristics^a^	Study design	Study duration (washout^b^)	Active intervention	Nitrate dose in active arm	Control	Impact on %FMD
Jones et al (2019)^20^	11/7^c^	Healthy, 65 ± 2.4/61 ± 1.9 y^c^^,^^d^	Parallel, RCT, double-blind	4 wk	BRJ	400 mg	Prune juice	↑
Burleigh et al (2019)^25^	11	Healthy, 100% M, 37 ± 2.1 y	Crossover, RCT, single-blind	8 d	BRJ	400 mg	NDBJ	↑
Sweazea et al (2018)^22^	23/22^c^	Healthy, 100% M, 24 ± 0.9 y/24 ± 9.4 y^c^	Parallel, RCT, double-blind	1 wk	Fruit and vegetable drink	280 mg	Prune juice	↔
Rammos et al (2014)^21^	11/10^c^	Mild CVD risk (mean heart score, 4.7), 63%/60% M, 63 ± 1.8 y/63 ± 1.3 y^c^	Parallel, RCT, double blind	4 wk	NaNO_3_	735 mg	NaCl	↑
Kapil et al (2015)^12^	32/32^c^	Hypertensive (mean BP 149/89), 50%/69% F, 58 ± 2.5 y/56 ± 2.9 y^c^	Parallel, RCT, double blind	2 wk (2 wk)	BRJ	375 mg (250 mL)	NDBJ	↑
Mayra et al (2019)^27^	10	Postmenopausal, 100% F, 52 ± 1.5 y	Crossover, RCT	10 d (2–3 wk)	High-nitrate salad	284 mg	Low-nitrate salad	↑
Velmurugan et al (2016) (2)^11^	34/33^c^	Hypercholesterolemic (mean, 6.7 mmol/L), 54%/57% M, 53 ± 1.8/53.3 ± 2.1^c^	Parallel, RCT, double-blind	6 wk	BRJ	375 mg (250 mL)	NDBJ	↑

↔ indicates no significant difference; ↑ indicates significant increase.

a Data are mean ± SEM.

b Where available.

c Data are presented for active/control arms.

d Gender split not known.

Abbreviations: BP, blood pressure; BRJ, beetroot juice; CVD, cardiovascular disease; F, female(s); FMD, flow-mediated dilation; KCl, potassium chloride; KNO_3_, potassium nitrate; M, male(s); NaNO_3_, sodium nitrate; NDBJ, nitrate depleted beetroot juice; RCT, randomized controlled trial.

Overall, 12 studies were conducted among healthy participants, and a remaining 5 studies were completed among participants who have high cholesterol^11^ (*n* = 2), HIV^30^ (*n* = 1), hypertension^12^ (*n* = 1), and abdominal obesity^26^ (*n* = 1). In terms of sex, 2 studies were conducted in only female participants,^27^^,^^28^ while 4 studies were completed among only male participants.^22^^,^^25^^,^^26^^,^^29^ In 1 study, male and female participants were assessed individually.^24^ Only 1 study was completed among pregnant participants.^28^ The mean participant age in the 5 chronic studies^11^^,^^12^^,^^20^^,^^21^^,^^27^ was more than 50 years, while only 2 acute studies^11^^,^^26^ were completed with older-aged participants. Except for 1 study,^26^ all other studies reported a BMI of less than 30 kg/m^2^. One study assessed both the acute and chronic effects of nitrate on FMD separately.^11^

Inorganic nitrate was usually given in the dietary form (*n* = 10), typically as beetroot juice versus a nitrate-depleted beetroot control (*n* = 10), but nitrate-rich vegetable juice^22^ and high-nitrate salad^27^ compared with prune juice and low-nitrate salad, respectively, were also used in 1 trial each. The nondietary sources of nitrate used were sodium nitrate (*n* = 3)^21^^,^^23^^,^^29^ and potassium nitrate (*n* = 2),^24^^,^^26^ while sodium or potassium chloride and/or water were used a placebo. The nitrate amount in the interventions varied from 225 mg to 1058 mg.

### Risk of Bias in Studies

According to the Cochrane RoB 2 tool, the overall bias of the most studies (∼60%) was classified as low risk. The remaining studies were classified as having some concern due to a lack of information about the randomization process and not providing detailed information on a prespecified analysis plan (Figure S1). Almost all reported trials were double-blinded (1 study was single-blinded^25^ and 1 study did not state the blinding process^27^).

### Results of Synthesis

**Figure 2.**
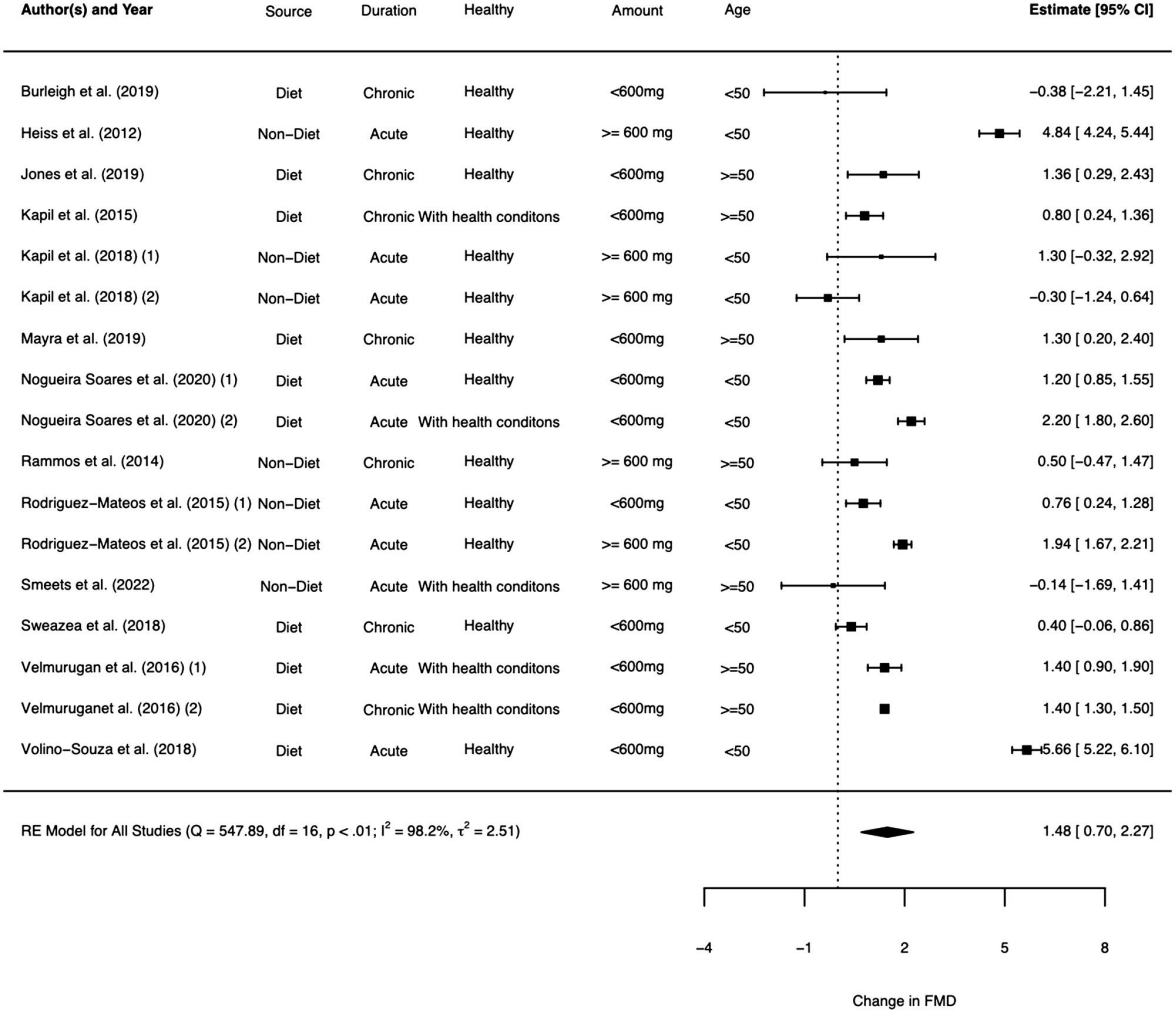
Forest Plot of the Studies Investigating the Effects of Inorganic Nitrate Supplementation on Endothelial Function as FMD. Summary estimate values are standardized mean differences for mean %FMD change before and after nitrate supplementation. Abbreviations: FMD, flow-mediated dilatation; RE, regression

The meta-analysis showed a significance in mean change in %FMD (SMD: 1.48%; 95% CI: 0.70%–2.27%; *P* < .01); high heterogeneity (*I^2^* = 98.2%) was observed (Figure 2). Subgroup analyses were performed in terms of inorganic nitrate dosage (low, <600 mg; high, ≥600 mg nitrate), type of the intervention (dietary and nondietary), participants’ health status (healthy and with a health condition), study duration (acute and chronic), and mean participant age (<50 or ≥50 years). Notably, in acute studies, nitrate intervention caused a much higher FMD increase compared with chronic studies (1.93% [95% CI: 0.71%–3.15%; *I^2^* = 98.4%] vs 0.90% [95% CI: 0.48%–1.31%; *I^2^* = 68.6%]), but with only moderate heterogeneity in chronic trials. Similar results were observed in participants aged 50 years or younger versus those older than 50 years (1.15% [95% CI: 0.84%–1.47%; *I^2^* = 45.7%] vs 1.81% [95% CI: 0.55%–3.08%; *I^2^* = 98.5%]). Subgroup analysis results are presented in Table 4.

**Table 4.** Estimates of FMD Change in Subgroup Analysis Based on Study Duration, Inorganic Nitrate Dosage, Intervention Type, and Health Status and Age of the Participants

Subgroup analysis	No. of studies	%FMD change, mean difference [95% CI]	*I^2^*, %
Study duration			
Acute	7	1.93 [0.71, 3.15]	98.4
Chronic	10	0.90 [0.48, 1.31]	68.6
Nitrate amount			
≥600 mg	6	1.41 [−0.17, 2.99]	96.2
<600 mg	11	1.51 [0.60, 2.43]	98.2
Health status			
Healthy	12	1.60 [0.52, 2.68]	89.8
With health condition	5	1.31 [0.72, 1.91]	97.7
Intervention type			
Dietary	10	1.59 [0.59, 2.59]	98.2
Nondietary	7	1.32 [−0.02, 2.66]	96.5
Age of the participants			
<50 y	7	1.81 [0.55, 3.08]	98.5
≥50 y	10	1.15 [0.84, 1.47]	45.7

Abbreviation: FMD, flow-mediated dilatation.

### Publication Bias and Sensitivity Analysis

Four sensitivity analyses were performed by removing different groups of studies (Table 5). For high-nitrate dosage, the Heiss et al^23^ study was removed as the control drink of the study included 1000 mg of nitrate. Another sensitivity analysis was carried out by substituting studies by Kapil et al^24^ (results from female participants), Burleigh et al^25^, and Smeets et al^26^ due to the high SD values of their FMD results. Furthermore, studies that used different control groups (prune juice^20^ and low-nitrate salad^27^) were excluded. Finally, studies by Heiss et al^23^ and Volino-Souza et al^28^ were substituted as these studies showed higher FMD responses compared with the other studies. Similar results were found after removal of these studies; the heterogeneity did not change.

**Table 5.** Outcome of Sensitivity Analysis

Reason for removal from analysis	Study (year)	FMD change, mean difference [95% CI]	Heterogeneity, *I^2^*, %
Nitrate dose >1000 mg	Heiss et al (2012)^23^	1.05 [0.68, 1.42]	89.2
Δ%FMD with 95% CI >1.5%	Kapil et al (2018) (1)^24^ Burleigh et al (2019)^25^ Smeets et al (2022)^26^	1.56 [0.69, 2.44]	98.4
Food-based control (other than nitrate-depleted beetroot juice)	Jones et al (2019)^20^ Mayra et al (2019)^27^	1.69 [0.82, 2.56]	98.6
Δ%FMD > 2 SDs from the mean	Heiss et al (2012)^23^ Volino-Souza et al (2018)^28^	1.05 [0.68, 1.42]	98.4

Abbreviation: FMD, flow-mediated dilatation.

**Figure 3.**
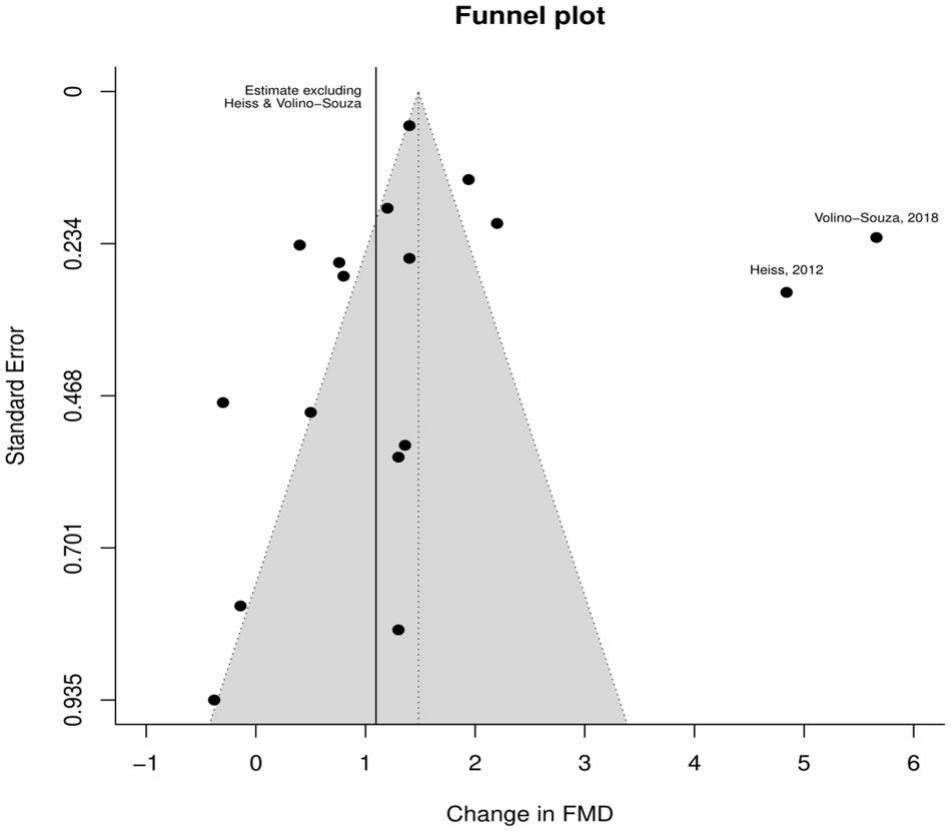
Funnel Plot to Evaluate Publication Bias of Trials Testing the Effects of Inorganic Nitrate on FMD Response. The solid line represents the impact of removing publications with a high FMD response (>2 × SDs from the mean). Abbreviation: FMD, flow-mediated dilatation

In the assessment of prospective studies of inorganic nitrate impact and FMD response, the point estimates were skewed slightly to the left of the weighted effect size, and distribution of the studies for FMD were not symmetrical, indicating potential publication bias. Substituting 2 studies that showed a higher FMD response compared with the other studies reduced the overall impact (Figure 3).

## DISCUSSION

A systematic review and meta-analysis of RCTs was performed to investigate the impact of inorganic nitrate on endothelial function. As a result, 13 included trials showed that inorganic nitrate consumption is associated with increased %FMD among adults: the pooled effect was 1.48%. Improvements in %FMD were also observed in subanalysis of both acute and chronic studies, inorganic nitrate dosage (<600 mg and ≥600 mg), type of intervention (dietary and nondietary), and participants’ age (<50 years and ≥50 years) in healthy and clinical participants. Improvements of the magnitude observed contribute evidence for the therapeutic potential for inorganic nitrate or the potential for dietary recommendations related to high-nitrate–containing foods for CVD prevention, although, admittedly, the high heterogeneity demonstrates inconsistencies in effect size. The effect size observed is comparable to that for flavan-3-ols,^31^ phytochemicals that now have a dietary recommendation due to their vascular benefits.^32^

Unlike previous systematic reviews that assessed the impact of nitrate on endothelial function, we used strict inclusion criteria to try and maximize the quality of the included trials and to minimize heterogeneity. We focused exclusively on macrovascular endothelial function as measured by FMD and excluded trials that assessed impact after ischemic insult. We also excluded trials that omitted measurement of baseline values of FMD and only included trials that assessed the change from baseline. Although this is the first comprehensive systematic review and meta-analysis where the primary objective was to assess the impact of inorganic nitrate on FMD response, 3 systematic reviews have previously assessed this in subanalysis with more modest results, although all supported our results demonstrating favorable effects: 0.42%, *P* = .002^33^; 0.59%, *P* < .001^34^; and 0.62%, *P* = .002.^35^ The reduced effect sizes observed with these reviews compared with ours could be related to the differences in inclusion criteria. First, none of the systematic reviews included trials that were published after 2020 and therefore omitted the work of Nogueira Soares et al,^30^ which showed a marked improvement with nitrate on FMD. Although we omitted 2 studies that included trials that assessed FMD after ischemia reperfusion (I/R) injury,^16^^,^^17^ these studies were included in other meta-analyses,^33–35^ which could have impacted their results. These studies demonstrated mean differences of approximately 0.5% FMD, which could have impacted the mean differences found. We chose to exclude these studies as they assessed mitigation of attenuation of FMD rather than improvements. Additionally, our decision to exclude trials that used mixed meals as a comparator could have impacted our findings. Similarly, to the I/R trials, these trials assessed the reduction in impairment (this time, postprandial in response to a high-fat meal) and therefore are difficult to interpret in the context of FMD effects. Nonetheless, of the 2 trials that were identified, 1 was not included in the previous systematic reviews.^16^

Although after subanalysis we observed an improvement in FMD in both acute and chronic trials, the increase was far greater in acute trials compared with chronic trials, although the heterogeneity was lower in the latter. Chronic trials are arguably more important for assessing the long-term health effects of a substance and provide a useful insight into the health potential of a substance. Unlike organic nitrate, inorganic nitrate is not considered to exhibit tolerance effects,^36^ and so this is unlikely to be the cause of the difference. Due to the small number of publications included in the each subanalysis, the differences are more likely to be due to intertrial variation; indeed, Velmurugan et al^11^ (the only study that was conducted acute and chronically) noted the same FMD response at both 3 hours post–beetroot juice consumption and after 6 weeks of beetroot juice consumption in older adults.

Inorganic nitrate interventions are commonly provided in both dietary and nondietary forms; these interventions were analyzed in the subanalysis. The subanalysis did not reveal vast differences in effect size between the 2 types of intervention (or in heterogeneity). Therefore, due to the negative associations with inorganic nitrate consumption and carcinogenic nitrosamine formation, it would be favorable to consume nitrate in vegetable form where vitamin C (an antioxidant) is also present, which has been demonstrated to prevent nitrosamine formation.^37^

Although in relation to BP reductions with nitrate, a dose-dependent response has been suggested,^7^ we observed no difference in %FMD increase in the high- versus low-dose stratification (we did not seek to assess the dose–response potential). Notably, the high doses in this review were considerably lower than in the review of BP (the highest dose here was 1028 mg vs 2790 mg in the BP review). One trial investigated the impact of different quantities of nitrate (3 mg/kg and 8.5 mg/kg body weight) and showed that, at 2 hours after ingestion, FMD response was higher in the high-dose group; however, after 4 hours, it reached the same level.^29^ Although ascertaining dose response was not an aim of this work, these data suggest that obtaining a high dose of nitrate may not be important to induce beneficial effects on endothelial function; there may be a dose threshold or optimal dose to induce benefits, which, indeed, has been suggested with other plant bioactives.^38^

There are limited data investigating the impact of nitrate on healthy participants compared with those with health conditions. In an attempt to assess this, a subanalysis was performed. Due to the vast breadth of health conditions (hypertensive, HIV-positive, pregnant, hypercholesterolemia, obese) and hence potentially large number of confounding factors (eg, medication) as well as the limited number of trials, it is hard to deduce any concrete conclusions from these data. The FMD responses were similar in the 2 groups, although, unsurprisingly, the heterogeneity was lower in the healthy subgroup. The use of nitrate in clinical populations warrants further investigation.

Subanalysis of the data with age of the participants dramatically reduced the heterogeneity, with the trials in older adults (≥50 years) demonstrating far less heterogeneity, although less improvement in FMD. The more similar hormonal profile of men and women postmenopause could be the reason for this, as it has previously been demonstrated that men and women respond differently to inorganic nitrate, potentially driven by the influence of sex hormones,^24^ although the majority of the RCTs included in the analysis were performed only in male participants.

### Clinical Implications

Given that a 1% increase in FMD is associated with a 13% reduction in the risk of adverse cardiovascular events,^14^ the pooled effect of an approximately 1.5% improvement observed in our analysis could correspond to nearly a 19.5% reduction in cardiovascular risk (assuming a linear relationship). The subgroup analyses further revealed that, while acute supplementation had a more pronounced immediate effect on FMD, chronic supplementation still provided substantial benefits, which may be more relevant for long-term cardiovascular protection. Notably, both lower and higher nitrate doses led to similar FMD improvements, suggesting that even moderate intake levels can be effective. Additionally, dietary interventions showed a slightly greater impact than nondietary sources, emphasizing the potential of natural food-based strategies. Although younger individuals exhibited a larger response, the consistency of effects in older adults suggests that dietary nitrates could be particularly beneficial in reducing cardiovascular risk in this at-risk group. These findings highlight the potential role of dietary nitrate supplementation as an accessible and cost-effective approach for CVD prevention, particularly in older or vulnerable populations.

### Limitations and Strengths of This Review

This is a comprehensive and up-to-date systematic review with meta-analysis that only focused on the impact of nitrate consumption on FMD response, the gold-standard measure of endothelial function. Unbiased, critical approaches were used and the data are up to date. However, it should be acknowledged that the depth of the review is dictated by the selected search terms. Although every effort was made to make the search as thorough as possible, there is a possibility that this could have led to trials being missed. Further, the omission of non–English-language publications could have led to missing trials. The data presented are only as good as the quality of data used and the quality of the trials included; and the availability of data will impact the results presented herein. Small sample sizes, short study durations, and usage of different control/placebo interventions and participant demographics are likely to have contributed to the high heterogeneity observed.

## CONCLUSION

Our systematic review and meta-analysis showed that beetroot juice and inorganic nitrate consumption resulted in a significant improvement in endothelial function measured by FMD. As brachial artery FMD is inversely related to future CVD events,^39^ this improvement suggests inorganic nitrate as a useful strategy for preventing future CVD. There is potential for preventative applications of inorganic nitrate and the data herein could be used to support dietary recommendations for nitrate-containing foods for a reduction in CVD.

## Supplementary Material

nuaf132_Supplementary_Data

## Data Availability

The data that support the findings of this study are available from the corresponding author upon reasonable request.
